# Eco-evolutionary feedbacks can rescue cooperation in microbial populations

**DOI:** 10.1038/srep42561

**Published:** 2017-02-13

**Authors:** Clara Moreno-Fenoll, Matteo Cavaliere, Esteban Martínez-García, Juan F. Poyatos

**Affiliations:** 1 Logic of Genomics Systems Lab (CNB-CSIC), Madrid, Spain; 2 BBSRC/EPSRC/MRC Synthetic Biology Research Centre, University of Edinburgh, Edinburgh, UK; 3 Molecular Environmental Microbiology Lab (CNB-CSIC) Madrid, Spain.

## Abstract

Bacterial populations whose growth depends on the cooperative production of public goods are usually threatened by the rise of cheaters that do not contribute but just consume the common resource. Minimizing cheater invasions appears then as a necessary mechanism to maintain these populations. However, that invasions result instead in the persistence of cooperation is a prospect that has yet remained largely unexplored. Here, we show that the demographic collapse induced by cheaters in the population can actually contribute to the rescue of cooperation, in a clear illustration of how ecology and evolution can influence each other. The effect is made possible by the interplay between spatial constraints and the essentiality of the shared resource. We validate this result by carefully combining theory and experiments, with the engineering of a synthetic bacterial community in which the public compound allows survival to a lethal stress. The characterization of the experimental system identifies additional factors that can matter, like the impact of the lag phase on the tolerance to stress, or the appearance of spontaneous mutants. Our work explains the unanticipated dynamics that eco-evolutionary feedbacks can generate in microbial communities, feedbacks that reveal fundamental for the adaptive change of ecosystems at all scales.

In many bacterial populations, resources produced by an individual may benefit other members of the population. These resources generally entail compounds secreted into the environment, which in effect work as public goods. Classical examples comprise molecules that can specify the precise density reached by a population (quorum-sensing autoinducers)^1^, support the assemblage of collective multicellular structures (biofilms extracellular polymeric substances)^2^, or process basic nutrients that would be unavailable otherwise (iron-scavenging siderophores, exoenzymes catalyzing the decomposition of complex sugars, etc.)^3^,^4^. Notably, the functions of these, and equivalent, public goods are constantly at the risk of being disabled by the invasion of cheaters in the population, individuals that do not contribute and only reap the advantages of the common resource^5^,^6^,^7^,^8^. The threat of cheaters represents at a microbial scale a well-known public-good dilemma, known as the tragedy of the commons^9^, and can fundamentally interfere with the sustainability of these communities. With their particular relevance to humans in matters of health (microbiome)^10^, and industry (bioremediation, biofuels, etc)^11^, the necessity of recognizing the consequences of social dilemmas in microorganisms becomes even more significant.

Much previous work focused therefore on identifying mechanisms that prevent the invasion of cheaters^7^. Here, we show how cheating can instead induce the continuance of cooperation, a prospect that has yet remained largely unexplored^6^. This is linked to the synergistic effects of spatial structure and eco-evolutionary feedbacks, that impact in a nonintuitive manner on the dilemma^12^,^13^. We considered specifically a scenario where a bacterial community is organized as a dynamical metapopulation (i.e., the community is transiently separated into groups, adding to other implementations of spatial structure such as groups connected via migration, and range expansions)^14^,^15^,^16^, and a public good is essential for its survival. Spatial structure is a well-known universal mechanism to promote cooperation^17^, which frequently emerges in bacterial populations, for instance, due to the restricted range of microbial interactions^18^,^19^. However, it is much less understood how the presence of structure affects the maintenance of cooperation when combined with explicit population dynamics (earlier work usually assumed constant population and only examined evolutionary dynamics)^20^.

The change in population size associated to the essentiality of the public good can indeed bring about complex eco-evolutionary feedbacks^12^,^13^,^21^, in which both population density –ecological dynamics– and frequency of cooperators –evolutionary dynamics– influence each other. The connection between these feedbacks and spatial structure has been theoretically described to allow the dynamical persistence of cooperation^15^ but largely remains an open problem that has started to be experimentally addressed only recently^22^,^23^,^24^,^25^. We show in this work how such connection can direct to the unexpected consequence that the population collapse linked to cheater invasions eventually generates conditions that contribute to the revival of cooperators. This represents, more broadly, an example of the effects that both ecological and evolutionary forces can generate on community dynamics when acting on similar scales^26^,^27^.

## Results

### Eco-evolutionary dynamics

**Figure 1:**
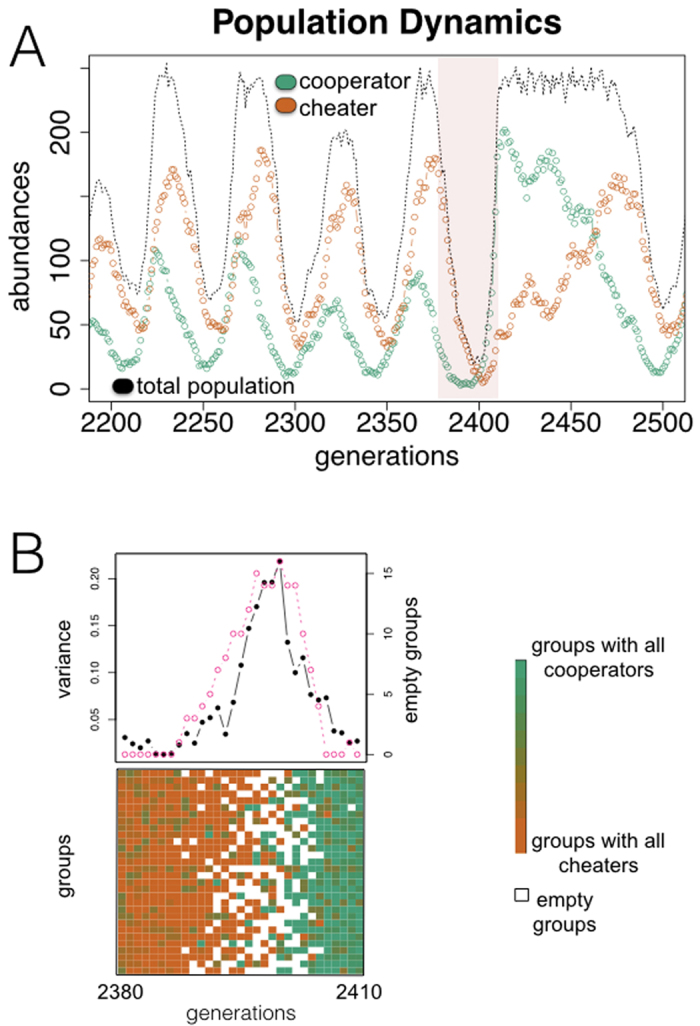
Exploitation by cheaters contributes to the rescue of cooperation. (**A**) Typical population dynamics obtained with an *in silico* model of a microbial community, which is organized as a transient metapopulation (Fig. S1) (Materials and Methods). Growth depends on an essential public good produced by the “cooperator” individuals. When “cheaters” invade, the decline in the amount of public good drives the collapse of the total population. This collapse paradoxically determines its subsequent revival. (**B**) Revival is coupled to the endogenous emergence of variability in the composition of the groups (constituting the metapopulation) when the population is falling^12^,^21^, and the following occurrence of groups only constituted by cooperators. For the time window highlighted in (**A**), we display group composition (bottom; ratio of cooperators on each group is colored according to the gradient shown; white squares denote empty groups, of a total of N = 30), intergroup diversity (top; black curve, quantified as variance in group composition), and number of empty groups (top; pink curve).

To analyze this scenario, we first introduced a stylized *in silico* model considering an initial finite population of agents –representing bacteria– with a given frequency of cooperators (producers of a public good, with a fitness cost) and cheaters (nonproducers, that could have emerged originally from the cooperators by mutation) (Materials and Methods). The population is temporarily organized in groups, where interactions take place (Fig. S1). These interactions are modeled by means of a public good game with individual reproduction being set by the game payoff^12^,^21^. Figure 1A displays a representative trajectory of the model: an increase of the cheating strain, due to its fitness advantage, causes a decrease in population density (less public good available). The demographic fall originates in the end variation in the composition of the groups, facilitating population assortment and the appearance of pure cooperator/cheater groups. Since the groups uniquely constituted by cooperators grow larger, they can ultimately reactivate the global population promoting again new cheater invasions. The whole process manifests in this way as a continuous cycle of decay and recovery of the community (Fig. 1A,B) (Materials and Methods). Demographic collapses consequently turn into an endogenous ecological mechanism that causes the required intergroup diversity, supporting the overall increase of cooperators −a mechanism that resembles a statistical phenomenon known as Simpson’s paradox^14^,^23^.

### Engineering of a synthetic bacterial community

**Figure 2:**
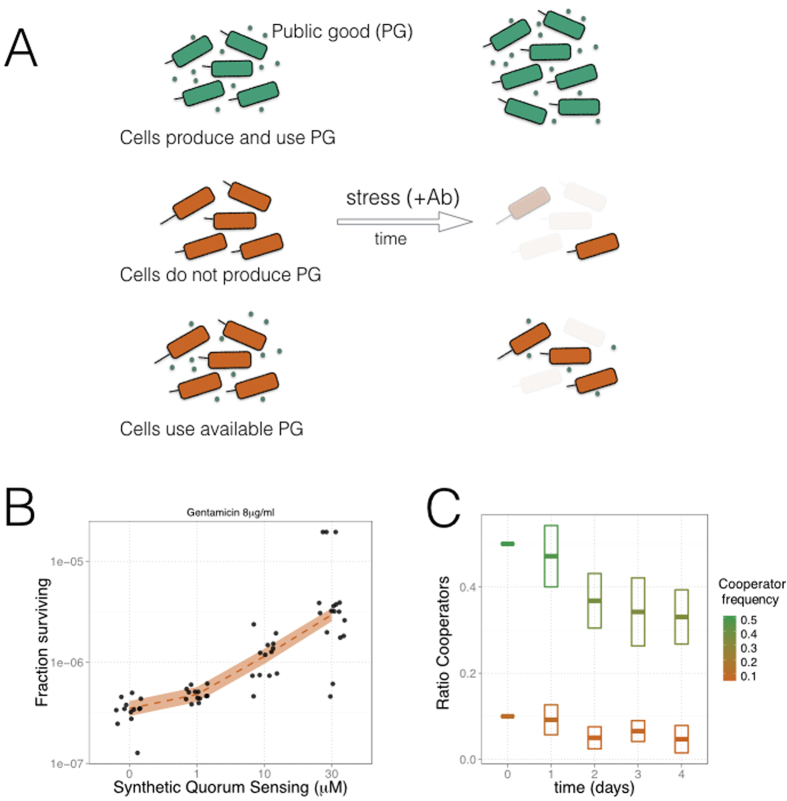
Engineering of synthetic strains that contribute (green), or not (red), to the production of quorum-sensing (QS) molecules acting as an essential public good (Fig. S2). (**A**) A cell community that accumulates (top, cooperators) or obtains (bottom, cheaters) the public good in the environment is able to tolerate a bactericidal antibiotic stress (gentamicin, Gm), although the latter can survive least because the initial public good is spent. Moreover, a community growing without public good would eventually collapse (middle). (**B**) Survival to Gm of a population constituted only by cheaters increases when the essential QS molecules are added to the preincubation medium, before growing under the stress (8 μg/ml Gm, N = 17 replicas per QS dosage, solid color represents 95% confidence interval of a local polynomial regression). (**C**) Fraction of cooperators in a mixed population decays with time due to the invasion of cheaters, which do not pay the cost of making the public good (Fig. S4). This is independent of the initial fraction (10% or 50% of cooperators). Boxes indicate standard deviation linked to the experimental estimation of ratios. See SI Materials and Methods for further details and protocols.

We then tested these ideas experimentally by engineering a synthetic public good interaction that is essential for the survival of a microbial population to a bactericidal antibiotic (Fig. 2A). Specifically, we constructed an experimental system in which a synthetic *Escherichia coli* strain (the cooperator/producer) constitutively expresses a diffusible autoinducer molecule acting as public good. This molecule is part of a quorum-sensing (QS) system foreign to *E. coli*, which includes a cognate transcriptional regulator. We connected this machinery to the expression of a gene that enables the synthetic strain to tolerate the bactericidal antibiotic gentamicin (Gm) [Fig. S2, the system is a variation of an earlier one^23^] (SI Materials and Methods). A second strain (the cheater/nonproducer) that only utilizes the public good can also be part of the community (we labeled the cooperative and cheater strains with a green and red fluorescent protein, respectively, to make possible population measurements) (SI Materials and Methods). Two crucial aspects distinguish in this way the designed setup. First, the presence of public good is an essential requirement to tolerate stress (Fig. 2B) (Fig. S3). Second, the system exhibits an intrinsic vulnerability, as cheaters could overtake the entire community by evading the cost of producing the public good (Fig. 2C) (Fig. S4). While in this case the presence of cheaters is part of the synthetic design, their emergence as result of mutations is well documented in natural settings^5^,^6^.

### Experimental eco-evolutionary dynamics

**Figure 3:**
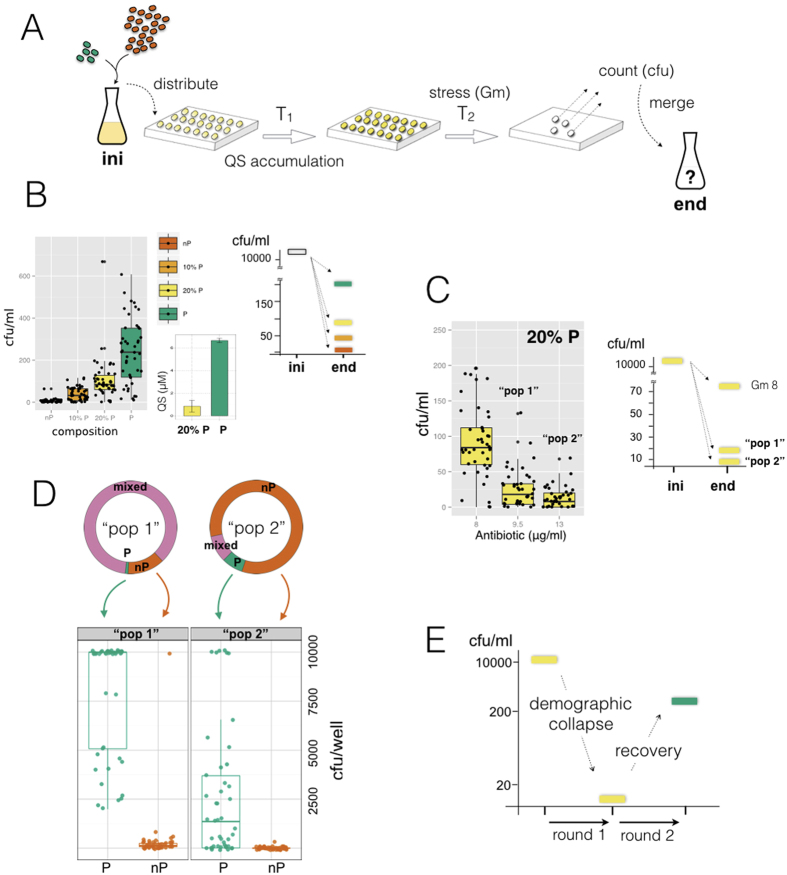
Experimental test of the eco-evolutionary feedback. (**A**) An initial population (ini) with specific cell density and composition is distributed in a multiwell plate. Groups (wells) grow and accumulate quorum-sensing (QS) molecules (T_1_, without antibiotic) and are reseeded into medium with gentamicin (Gm) (T_2_). The final population is quantified by plating each well and merging the values to estimate the full population (end). (**B**) Demographic collapse after exposure to 8 μg/ml of Gm of populations with different compositions (represented by colors) (Left panel). Dots represent the result of groups within a metapopulation, box plots represent associated statistical parameters (N = 45). Cooperators/cheaters labeled as producers (P)/nonproducers (nP) of public good, respectively. (Inset). Accumulation of QS at T_1_ in groups constituted by 20% or all Ps. (Right panel). Demographic decline of the full population as a function of composition. (**C**) (Left panel). Demographic collapse under three Gm dosages of groups within a metapopulation from initial density as (**B**) and 20% Ps. Dots and box plots also as in (**B**). (Right panel). Demographic collapse of the full population as a function of Gm. Population densities, and associated dosages of 9.5 μg/ml and 13 μg/ml are correspondingly termed as “pop 1” and “pop 2” conditions. (**D**) In a second round of the experimental protocol the initial conditions correspond to those of either “pop 1” or “pop2” in (**C**). The pie charts show the experimental distribution of nonempty wells. Quantification of the characteristic tolerance of groups of only Ps and only nPs is done by engineering replica populations with “pop 1” and “pop 2” cell densities, application of the protocol with correspondent Gm, and measurement of the recovery (dots represent a replica well, N = 45). A maximal value of ~10000 cfu/well denotes strong recovery. (**E**) While the first round of the protocol causes a demographic collapse of the initial full population (20% Ps), it also generates a sufficient variance in the metapopulation, in a second round, to allow the recovery of cooperators (recovery is proportional to the characteristic growth of P wells and their abundance in a “pop 2” metapopulation, see Materials and Methods). This confirms the eco-evolutionary feedback.

The experimental validation of the presented eco-evolutionary feedback aims to capture its two most important features: the demographic collapse induced by cheaters, and the subsequent recovery of cooperation supported by the spatial constraints. This is done by using a minimal experimental protocol that approximates the dynamics of the computational model (Fig. 3A). The protocol starts with the distribution of an initial bacterial community into a metapopulation (multiwell plate), with each well growing and accumulating public good for a given time. The metapopulation is then exposed to stress (Gm). The resultant densities of cooperators and cheaters of each group are quantified by plating (viable colony counting, Materials and Methods) and the outcome is merged in order to determine the collective dynamics of the community. The protocol is in this way intentionally designed to reproduce the transiently structured interaction between cooperators and cheaters (equivalent to the random assortment into groups in the model, Fig. S1). Moreover, each output of a single round can become the new initial condition for a prospective next round.

**Figure 4:**
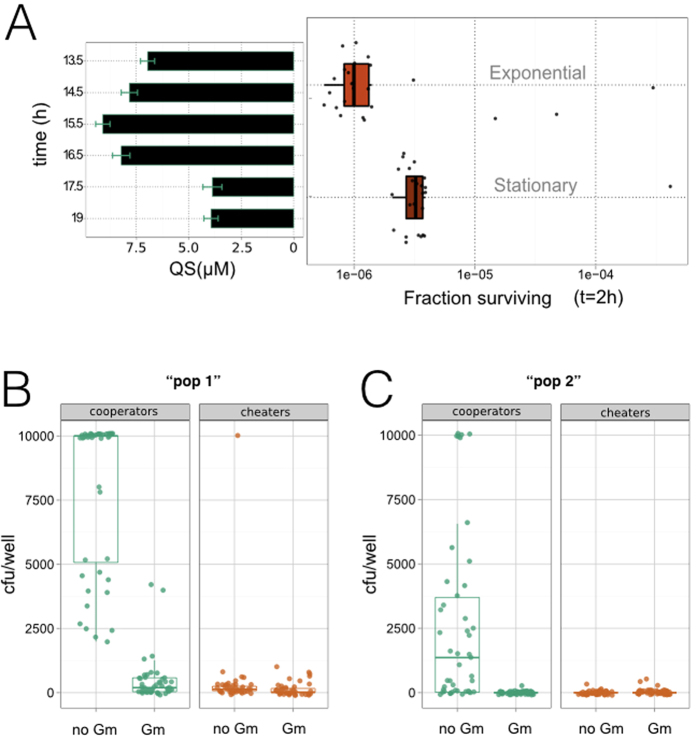
Influence of secondary effects on the maintenance of public good-based communities under antibiotic stress. (**A**) Cheater cells in exponential or saturating phase were resuspended in medium with gentamicin (Gm) (Materials and Methods). (Right) Cells in exponential phase experienced less antibiotic tolerance (Gm = 12 μg/ml, dots correspond to N = 45 replicas, box plots indicate associated statistical parameters). (Left) Concentration of quorum-sensing (QS) molecules decays as a function of time of growth (Byers *et al*.^28^) (Fig. S9), starting from an initial low-density of cooperators, i.e., “pop 2” density condition in Fig. 3. Bars represent measurement errors associated to QS estimation (Methods). Starting from these “pop 2” initial densities, and after an accumulation time of 15.5 hrs (T_1_), cells are in exponential phase, and the amount of QS is maximal. Recovery is thus strongly linked to the presence of the public good. (**B**,**C**) Emergence of spontaneous mutants to Gm. Initial populations of cooperators and cheaters are subjected to an accumulation and stress protocol under the “pop 1” and “pop 2” conditions (same as Fig. 3; black dots represent replicas, N = 45; box plots represent statistical parameters, color codes as Fig. 3). We repeated the experiment for each strain and dosage, so that one can quantify the typical resulting population, and also the mutant subpopulation (by plating with Gm and without no Gm antibiotic; the specific plating dosage corresponds to that of the matching growing conditions). Emergence of spontaneous mutants is reduced at higher dosage (**C**), i.e., “pop 2” conditions. Tolerance is most significantly associated in this regime to the presence of the public good.

We examined first the demographic collapse. Both the *in silico* model and the main attributes of the experimental system predict that a community with different frequency of cooperative and noncooperative strains would accumulate a distinct amount of public good, and thus present different tolerance to stress. To confirm this, we engineered an initial population with density ~10^4^ cells/ml and different composition (Fig. S5), and distributed it as a metapopulation (SI Materials and Methods). Figure 3B confirms the demographic collapse of each well according to composition (left panel; associated inset illustrates the corresponding range of accumulation of QS molecules), while also illustrating the differential collapse of the whole population (right panel). The large initial density ensures homogeneity between the groups when distributed what de-emphasizes spatial effects. Some outliers here are associated to the emergence of spontaneous mutants, see Fig. 4. In this way, the behavior of the full population is equivalent to that of a single group, i.e., every group represents in practice a replica of the same conditions. Moreover, rising the strength of stress, i.e., essentiality of the public good, also impacts on collapse (Fig. 3C, fixed composition of 20% P but increasing antibiotic dosage). Note that the fraction of producers in the collected final populations does not vary substantially in this regime (Fig. S6).

We followed the fate of two of the previous trajectories to confirm how the feedback from population dynamics can cause the recovery of cooperation. Populations with 20%Ps under medium (9.5 μg/ml) and strong (13 μg/ml) Gm stress exhibited, in the first round, an increasing demographic decline (Fig. 3C; in what follows, we labeled them as “pop 1” and “pop 2”). We expect these declined densities to give rise to high composition variance in a subsequent round of the protocol (similar to the model, Fig. 1B). Indeed, an initial population with “pop 1”, or “pop 2”, density and composition generated high variance when allocated into a metapopulation, as in Fig. 3A, but with drastically different distributions of group composition (experimental distributions of nonempty groups illustrated in the pie charts of Fig. 3D) (Fig. S7). The population that exhibited the higher collapse in round 1 (“pop 2”) generates groups with very few cells and strong assortment of cooperators, which is the group class best equipped to tolerate stress. Both metapopulations were later subjected to bactericidal stress (that is, medium and strong Gm dosages) followed by quantification.

The outcome of this second round is influenced by high variability in the response to the intense antibiotic stress, combined with the scarce presence in the resultant metapopulation of some of the groups (Fig. 3D, top pie charts). While recovery was confirmed with tested examples of explicit metapopulations (“pop 2” metapopulations individually traced to the end of the second experimental round, Fig. S8), we more comprehensively characterized the fate of “pop 1” and “pop 2” metapopulations in a complementary way. We chose to measure the behavior of a large number of pure cooperator and pure cheater groups (best case and worst case scenarios in terms of stress tolerance, respectively) under the corresponding conditions of population density and Gm stress. The expectation that cooperator groups recover disproportionately is observed consistently under this analysis (Fig. 3D, bottom). Therefore, when all the groups of a “pop2” metapopulation are pooled together, cooperators typically increase in frequency and population density recovers (Fig. 3E), in what is a manifestation of the eco-evolutionary feedback. Hence, the demographic decay of the population caused by the invasion of cheaters reveals crucial to attain the necessary heterogeneity among the various groups, and allows the recovery of cooperation.

### Secondary effects on the dynamics

Could other features intrinsic to the biological system under consideration modify the previous dynamics? We explored this issue by first describing two constraints linked to the specific experimental model, and then evaluating their potential consequences for the recovery. The first one identifies a possible trade-off between the build-up of public good and the growth stage of the population before exposure to the antibiotic. As Fig. 3B illustrates, the more public good a population accumulates (i.e., the longer it grows), the more it tolerates stress. However, extensive growth often implies a deterioration of the environmental conditions (that is, of the growth medium), which could cause the public good molecule to lose its functional activity^28^ (Fig. 4A) (Fig. S9). Growing too much could be problematic. Moreover, when a bacterial population stays some time in saturating conditions, before resuming growth, it presents a longer lag phase that indirectly protects bacteria from antibiotics^29^, independently of whether public good is available or not (Fig. 4A). Within the previous context of the revival due to the eco-evolutionary feedback (Fig. 3C,D, “pop2” conditions), low initial densities assure a regime further from saturation at the end of the accumulation period. This would imply less decay of the public good and shorter lag, i.e., the dynamics is not strongly altered by the previous aspect and does become mostly associated to the action of the public good.

The second constraint relates to the emergence of spontaneous mutants, which can resist the antibiotic and enable the survival of cheater populations in the absence of public good^30^. Spontaneous mutation acts as an additional resistance mechanism to the antibiotic, also representing a constraint on the duration of growth and size of the initial/final populations (and thus on the accumulation of public good): a larger initial population increases the chance for a mutant to arise (Fig. 4B,C). This type of rescue becomes more frequent as the antibiotic dosage is decreased^31^ (Fig. S10). Of note, both of these factors are less significant when the collapse of the population is very strong (“pop2” situation involving smaller initial population size, and stronger dosages), that is when the best conditions exist for the eco-evolutionary feedback. Overall, in the specific regime where our synthetic system reveals the feedback, secondary factors are not expected to considerably modify the eco- evolutionary dynamics.

## Discussion

This study emphasizes that the combination of spatial constraints and specific attributes of the public good (essentiality) can be crucial for the outcome of the eco-evolutionary dynamics in cooperative bacterial communities. This is important in circumstances where the public good aids tolerance to stress, when nonintuitive effects may appear: an increase in cheaters frequency, or stress intensity, can actually precede the recovery of cooperation in the population. The particular experimental setup also provides synthetic strains with essential public goods. While in other studies the absence of public good prevents growth of the population^23^, in our case it means the active killing of cells. These strains allow then to test the effects and the mechanisms of a “tragedy of the commons” (spreading of cheaters leading to a population collapse), a notion often mentioned in microbial cooperation, and indeed fundamental to this discussion. While the phenomenon we describe –cooperator preservation mediated by inter-group variability– shares some aspects with the Simpson’s Paradox (in which cooperators globally increase in frequency despite their local decay), we introduce one crucial feature. A Simpson’s Paradox can be achieved by establishing the required conditions (i.e. heterogeneity in group composition) *ad hoc* [e.g., by means of an external dilution-like perturbation^23^]; instead, we present an endogenous mechanism that provides composition variance and ensuing perpetuation of the cooperative behavior: essentiality of the public good directly ties the abundance of cooperators to population size after exposure to stress. Spatial structure translates the lack of population resilience due to cheater invasions into assortment of cooperators, and thus recovery. These basic properties of a biological system generate intrinsic eco-evolutionary dynamics that preserve cooperation.

Previous experimental work explored the role of frequency^4^,^32^,^33^, density^34^ or spatial structure^22^,^35^,^36^,^37^ dependencies, and also of multilevel selection^38^, in the evolution of cooperation, while the notion of eco-evolutionary feedbacks in microbial social systems is itself very new^25^. The specific integration of structure and eco-evolutionary feedbacks, with the added crucial aspect of public good essentiality, is however unprecedented. Furthermore, the idea of cheaters as integral part of a cycle that make conditions amenable to the continuation of cooperation in the long term, instead of necessarily disrupting it, is quite novel. Exceptions are, for instance, their possible contribution to the evolution of life cycles^6^, or stochastic cheating as a strategy to preserve cooperative virulence^39^. The novelty of our study lies in the combination of the counter-intuitive effect with the generality of the underlying principle, which can be extended to include more diverse species, e.g., those that could incorporate means of deceit signaling to exploit the social dilemma^40^. It is also worth mentioning that while we use very established theoretical concepts and a synthetic system, we tried to go beyond this restricted framework by incorporating the influence of other unrelated features of the biological system (growth phase, spontaneous mutants), hinting at the complexity of our general proposed phenomenon as it would play out in nature. In addition, the strategy of constitutively producing the public good –that we considered here to exemplify the phenomenon in the simplest manner– could be extended to more complex regulatory mechanisms commonly found in microbes^41^, with potentially interesting consequences^12^.

Moreover, the use of synthetic communities to analyze eco-evolutionary feedbacks further extend earlier applications to this aim of artificial communities constructed with natural species with no history of interaction^42^. Synthetic communities emerge therefore as tractable experimental models (“ecosystem simulators”) in which to begin to understand the tight ecological and evolutionary feedbacks increasingly observed in ecosystems worldwide^26^,^27^,^43^ by allowing explicit and precise manipulation of relevant properties of the organisms.

## Materials and Methods

### Ecological public good model

We used a model first described in ref. 12 to simulate the dynamics of a population whose growth is based on an essential public good. It is based on a one-shot public good game^44^ in which agents can contribute (cooperators) or not (cheaters) to the public good in groups of size N. Contributing implies a cost c to the agents. Group contributions are then summed, multiplied by a reward factor r (that determines the efficiency of the investments and the attractiveness of the public good) and redistributed to all group members, irrespective of their contribution. The public good game is characterized by the parameters N, r and c (group size, efficiency and cost of the public good, respectively, where we fixed c = 1 without loss of generality). Every simulation starts with an initial population constituted by a common pool of k identical agents in the cooperator state, where k is the maximal population size (carrying capacity), to be updated in a sequential way as follows (see also Fig. S1): (i) The common pool is divided in randomly formed groups of size N (i.e., N is the total number of individuals and empty spaces in each group). The number of formed groups is then └ k/N┘. (ii) In each one of the (non-empty) groups, a one-shot public good game is played. This means that cheaters receive the payoff P_cheater_ = icr/(i + j), while cooperators receive the same payoff minus a cost, i.e., P_cooperator_ = P_cheater_ − c; with i, j being the number of cooperators and cheaters in the group, respectively, and i + j ≤ N. After the interaction the grouping of individuals is dissolved. (iii) Each individual can replicate (duplicate) with a probability that is calculated by dividing its payoff by the maximal possible one (i.e. the payoff obtained by a cheater in a group of N − 1 producers). Each cooperator that replicates generates an offspring that is either a cheater (with probability ν) or an identical cooperator (with probability 1 − ν). (iv) Individuals are removed with probability δ (individual death rate). In simpler words, the life cycle of the computational model is characterized by two distinct stages. In stage I (steps i-ii), the population is structured in evenly sized randomly formed groups in which the public good game is played. In stage II (steps iii-iv, after groups disappear), each individual replicates according to the group composition (and payoff) experienced in stage I. Replication can happen only when the current total population is less than the maximal population size, k, i.e. there exists empty space (empty spaces are calculated by considering k minus the current amount of individuals in the population). If more individuals could replicate than the available empty space, only a random subset of them ultimately replicates (of size the number of empty spaces available).

### Experimental procedures

Initial populations were typically prepared by mixing cooperators and cheaters at a defined population density (in the required volume to fill a multiwell plate, to obtain 10^4^ cells/well for high initial density experiments and 1–10 cell/well for low initial density experiments) and cooperator frequency. After the accumulation of public good for 15.5 hrs (T_1_, grown in LB broth at 30 °C with constant shaking), and reseeding 1/10 to grow with stress for 8.5 hrs (T_2_, LB broth and specific Gm dosage at 30 °C with constant shaking), population size was quantified by spreading cultures onto 1.5% (w/v) agar plates with five 3mm glass beads for 30 s. These cultures were incubated at 30 °C for 48 h (or otherwise indicated). Then, to quantify the cell number of a population we counted colony forming units (cfu) under blue light illumination (LED transilluminator, Safe Imager^TM^ 2.0, Invitrogen, Waltham MA USA). Note that cooperators and cheaters were labeled with a GFP and mCherry fluorescent protein, respectively. We estimated the recovery of cooperators in Fig. 3E by considering the response of a representative “pop2” metapopulation with 4 Ps, 45 nPs, 5 mixed and 42 empty groups; with the only nonzero response being that of P groups (typical response is 1360 cfu, Fig. 3D, see also Fig. S8). The density after the second round of the protocol in a plate with 200 μl per well is then 4 × 1360/19.2 ml = 283.3 cfu/ml. A more detailed description of the experimental protocols including growth conditions, strain constructions, quantification of cheaters, and antibiotic sensitivity assays is given in SI Materials and Methods.

## Additional Information

**How to cite this article**: Moreno-Fenoll, C. *et al*. Eco-evolutionary feedbacks can rescue cooperation in microbial populations. *Sci. Rep.* **7**, 42561; doi: 10.1038/srep42561 (2017).

**Publisher's note:** Springer Nature remains neutral with regard to jurisdictional claims in published maps and institutional affiliations.

## Supplementary Material

Supplementary Information
