# Supplementary material for: Eco-evolutionary feedbacks can rescue cooperation in microbial populations
Source: Sci Rep. 2017 Feb 13;7:42561. doi: 10.1038/srep42561 (PMC5304172; doi:10.1038/srep42561)
Supplement: Supplementary Information [file srep42561-s1.pdf]

Supplementary Information for

Eco-evolutionary feedbacks can rescue cooperation in microbial populations

Clara Moreno-Fenoll[1], Matteo Cavaliere[1,2], Esteban Martínez-García[3], Juan F. Poyatos[1*]

[1]Logic of Genomics Systems Lab (CNB-CSIC), Madrid Spain

[2]University of Edinburgh, Edinburgh, UK

[3]Molecular Environmental Microbiology Lab (CNB-CSIC), Madrid, Spain

[*]Correspondence to: jpoyatos@cnb.csic.es

This PDF includes experimental Materials and Methods, and Figs. S1 to S10

Supplementary Materials and Methods

Media, growth conditions and chemicals

Strains were grown in LB broth (10g/l casein peptone, 5g/l yeast extract, and 5g/l NaCl) at 30°C with constant shaking. Overnight cultures were grown aerobically in flasks (170rpm) at 30°C, and experiments were performed in 96-well plates (Thermo Scientific, Denmark), with 200μl of medium, 50μl of mineral oil (Sigma-Aldrich, MO, USA), and shaking at 10000rpm and 30°C. Where indicated, antibiotics were added to the liquid medium or plate at final concentrations: kanamycin (Km) 50μg/ml, spectinomycin (Sp) 50μg/ml in the construction of strains and 25μg/ml in experiments, and/or gentamicin (gm) with concentration as noted in the experiments. The synthetic *quorum-sensing* molecule N-butyryl-L-homoserine lactone (C4-HSL) was purchased from Cayman Chemical (Mi, USA). Cell dilutions were done in PBS (pH 7.4, 80.6mM Na_2HPO_4, 19.4mM KH_2PO_4, 27mM KCl, 1.37M NaCl at 10X, USB Corporation, OH, USA).

Measurement of population size

Cultures were spread onto 1.5% (w/v) agar plates with five 3mm glass beads for 30s, and are incubated at 30°C for 48h (or otherwise indicated). Then, to quantify the cell number of a population we counted colony forming units (CFU) under blue light illumination (LED transilluminator, Safe Imager™ 2.0, Invitrogen, Waltham MA USA). The OD_{600} of cultures was measured in a VICTOR2x 2030 Multilabel Reader machine (Perkin Elmer, Waltham, MA, USA) with intermittent orbital shaking. For experiments with P and nP strains, Km and Sp were added to the media.

General DNA techniques, plasmids and strain constructions

The different *E. coli* strains were derivatives of JC1080 (BW25113 *Δsdi::FRT*)*[1]*. Oligonucleotides used in this work are indicated in Table S1. Plasmid DNA was prepared using the QIAprep Spin Miniprep kit (Qiagen, Inc., Valencia, CA). When required DNA was purified using the NucleoSpin Extract II (Macherey-Nagel, Düren, Germany). Colony PCR was performed by transferring cells directly from fresh agar plates into PCR reaction tubes.

The pZS4int-rhlL-GmLAA plasmid was constructed by PCR amplifying the *aacC1* gene (Gm^R), from pSEVA611 plasmid *[2]*, to which 33-bp, encoding the AANDENYALAA protease degradation tag was added to the 3′-end using primers Gm-kpnI-F and GmLAA-hindIII-R (Table S1). The ~0.5-kb PCR DNA was digested with KpnI and HindIII and used to replace the ~0.7-kb fragment containing the *cat*LVA resistance marker from pZS4int-rhl-catLVA *[1]* generating the pZS4int-rhlL-GmLAA plasmid. Thus, the "Genomic insert" parental strain was constructed by integrating the cassette T_0-Sp^R-*rhlR*←P_{lacIq}-P_{rhl}→Gm^R_{LAA}-T_1 into the λ attachment site (*attB*) of *E. coli* JC1080 with the helper plasmid pLDR8, that bears the lambda integrase, as described *[3]*. The pZS4int-rhlL-GFP plasmid was

assembled by extracting the *gfp* gene from pSEVA241-P_{rhl}→*gfp* vector (lab collection) upon digestion with KpnI and HindIII. Then, the 0.7-kb DNA fragment was cloned into the KpnI and HindIII sites of pZS4int-rhIL-GmLAA replacing the Gm^{R}_{LAA} cassette for a GFP fluorescent reporter.

Then, the quorum sensing reporter "biosensor" strain was constructed by introducing into the λ attachment site (*attB*) of *E. coli* JC1080 the cassette T_0-Sp^R-*rhlR*←P_{lacIq}-P_{rhl}→*gfp*-T_1 as described above. The plasmid pZS*2R-GFP-rhlI (ori-SC101*, P_R→*gfp-rhlI*, Km^R) was obtained from *[1]*. The pZS*2R-mCherry (ori-SC101*, P_R→*gfp-rhlI*, Km^R) plasmid was constructed by amplifying *mcherry* from pSEVA237R *[4,5]* using primers mCherry-kpnI-F and mCherry-xbaI-R (Table S1). The PCR-amplified fragment was flanked with KpnI and XbaI restriction sites and cloned into the corresponding sites of the ~3.5-kb pZS*2R-GFP-rhlI plasmid backbone, thereby swapping the *gfp-rhlI* cassette for the *mcherry* gene.

GFP expression assay to estimate QS signal concentration

To estimate the concentration of the quorum sensing signal produced (C4-HSL), in different experimental conditions we used the reporter "biosensor" bacteria. We grew the "biosensor" strain overnight, adjusted the OD_{600} to 0.1 and grew aerobically for 2.5h at 30°C. Then, the culture was aliquoted and purified supernatant from the experimental sample was added. We used known concentrations (0, 0.1μM, 1μM, 10μM, and 100μM) of the commercial N-butyryl-L-homoserine lactone (C4-HSL) to obtain a calibration curve. We grew these preparations aerobically for 3h, laid out 200 μl of cultures in quadruplicates in a 96-well black/clear bottom microtiter plate (Sigma-Aldrich, MO, USA) and measured OD_{600} and GFP fluorescence (ex: 488nm; em: 520nm; cutoff: 495nm) in a SpectraMax M2^e microplate reader (Molecular Devices, CA, USA). Supernatants were obtained from 200μl of overnight liquid cultures prepared following the "Accumulation of PG and stress" protocol, centrifuged twice at room temperature to remove cells and used directly to induce growing cells of the reporter strain. Comparing observed supernatant fluorescence to the calibration curve approximated QS molecule quantities.

Engineering of initial conditions

Initial populations for the "accumulation of PG and stress" protocol and others were prepared by mixing cooperators and cheaters at a defined population density (10^4 cells/well for high initial density experiments and 1-10 cell/well for low initial density experiments) and cooperator frequency. Overnight cultures of producers and nonproducers were washed twice with PBS by centrifugation for 15min at 3800rpm and room temperature. Then, OD_{600} was adjusted to 0.15. We assembled populations at the desired P frequency in a fixed final volume (2.5ml), which was then serially diluted to the required cell density. This dilution was done in large volumes of medium (20ml) and applying low dilution factor (¼) each step to minimize the introduction of error in strain frequencies. Initial dilution steps were performed in PBS and the final 3 steps were performed in LB with Km, Sp. The robustness of this procedure is shown in Fig. S5.

Invasion of nonproducers

We prepared washed cultures of producers and nonproducers as described above. After adjusting OD_{600}, to 0.15 we mixed both strains at the indicated frequency. Then, we inoculated three replica 50ml Erlenmeyer flasks with 5ml of LB, Km, Sp. After 24h, we reseeded a new flask with a 1/100 dilution and fresh medium. In order to estimate producer frequency in grown cultures, cells were 1/10 serially diluted in PBS using a total of 10ml of medium, plated onto LB agar plates, and colonies counted after 24h at 30°C. We followed this process for 4 consecutive days.

"Accumulation of PG and stress"

Populations with a given initial cell density and cooperator frequency were prepared, distributed into a 96-multiwell plate and incubated for 15.5h (T_1). Then, 1/10 of each well was transferred into a new 96-multiwell plate with LB, Km, Sp and the specified gm concentration. This plate is again incubated for 8.5h (T_2). At the end of T_2 we plated whole well content on LB agar plates and counted CFU after 48hrs at 30°C.

Antibiotic sensitivity

Overnight cultures were reseeded and grown for 4h at 30°C to reach exponential phase. Aliquots of this culture containing ~10^6 cells were resuspended into a 96-multiwell plate with LB and a given dose of gm, incubated for 2h or 4h, plated and counted. For experiments with the nP strain, Km and Sp were added to the LB medium. For experiments with cells in stationary phase, overnight cultures were used directly in the initial inoculation. We used additional wells without antibiotic in parallel to obtain a reference population size. We express the sensitivity to the antibiotic as "fraction surviving" (population size after exposure to antibiotic / reference population size without antibiotic).

Effect of synthetic quorum-sensing on gentamicin tolerance

Overnight cultures of nonproducers were resuspended into LB with Km, Sp and the indicated concentrations of synthetic *quorum-sensing* molecule (C4-HSL). Then, we proceeded with the antibiotic sensitivity assay as described above.

Mutation rate

We generated replica populations of the nP strain and initial density of ~1 cell/well. We distributed these cultures in 96-multiwell plates and allowed them to grow for 15.5h (T_1). We plated the whole well content on LB agar plates with the specified gm dosage and counted viable cells. From the distribution of gm^R CFU observed in a set of replica populations the mutation rate was estimated with a maximum likelihood method as described in *[6]*, using the online application "FALCOR: Fluctuation Analysis Calculator" (http://www.keshavsingh.org/protocols/FALCOR.html)

Supplementary Figures

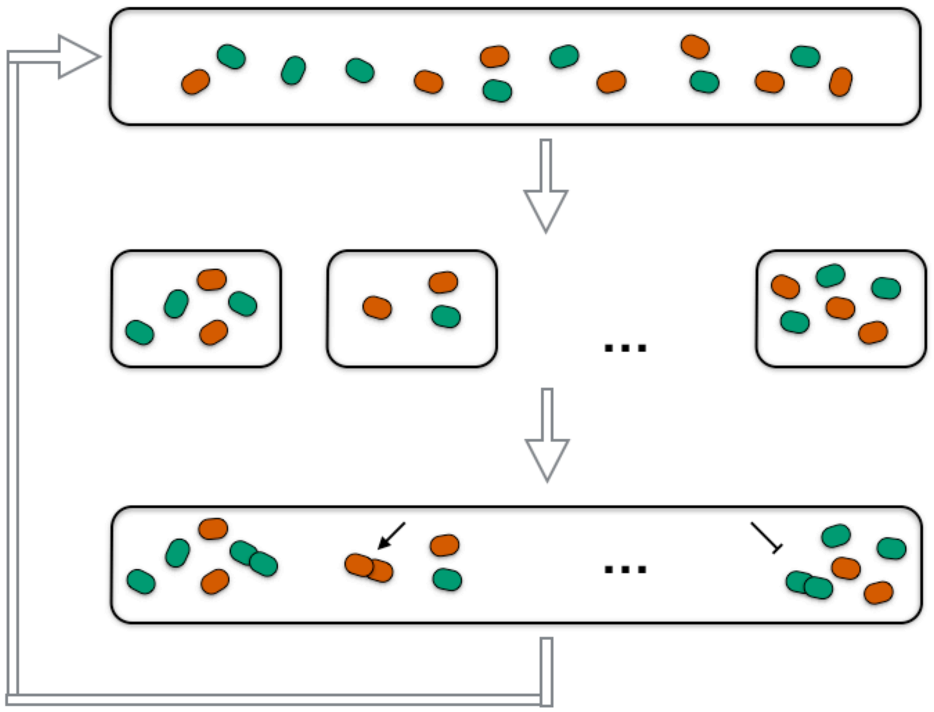

Figure S1. Ecological public goods simulation. A model first described in *[7]* was used to trace the dynamics of a population based on public goods. In these simulations, a population with a carrying capacity *k* is created with a given proportion of cooperators/producers (green ovals) and cheaters/nonproducers (red ovals). In each time step, this population is randomly assorted into groups of size *N*. In each group, the public goods game is played and fitness is assigned to each individual based on its identity (with an additional cost of being a producer) and the number of producers in the group. The individuals are pooled together again, and then divide (arrow) according to the calculated fitness and die (blunt arrow) with a fixed probability.

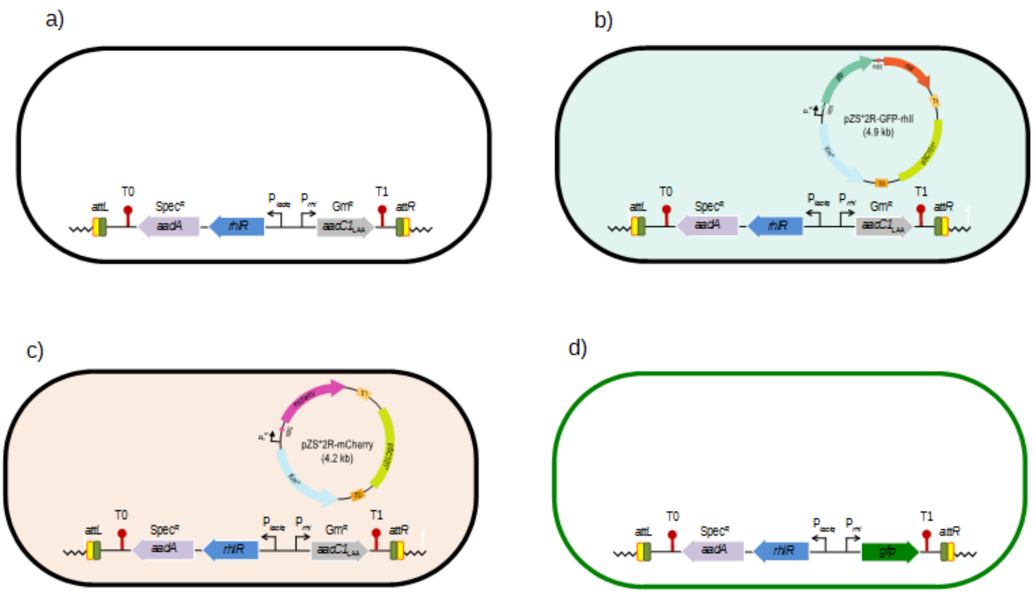

Figure S2. Characteristics of the engineered bacterial strains. The experimental system is based on two types of constructed bacterial strains: cooperators (producers) and cheaters (nonproducers). **(a)** They both possess a common genomic insert, which allows for recognition of the public good signal (transcriptional regulator RhlR) and expression of gentamicin resistance (gene *aacC1* under the promoter P_{rhl}). **(b)** The producer strain contains a plasmid enabling synthesis of the public good molecule (*rhlI,* that is, the *quorum-sensing* molecule N-butyryl-L-homoserine lactone or C4-HSL), and a GFP fluorescent reporter. **(c)** The nonproducer strain carries an equivalent plasmid with an mCherry fluorescent reporter and without the genes for public good production. **(d)** An additional "biosensor" strain allows us to quantify the amount of public good in the medium. In this strain, the public good molecule activates a GFP reporter instead of the antibiotic resistance gene.

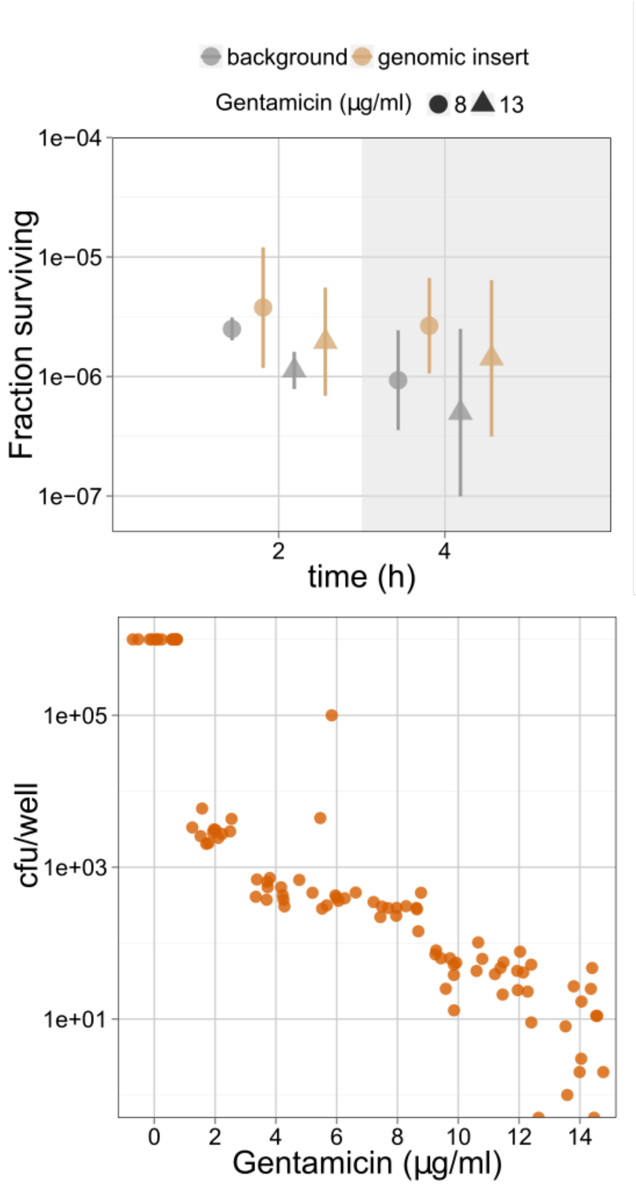

Figure S3. Antibiotic sensitivity of the strains. (Top) The genetic modifications considered include the insertion into the bacterial genome of a gentamicin resistance gene under a *quorum-sensing* inducible promoter. This might translate into fairly reduced sensitivity to the bactericidal. Thus, we tested the antibiotic sensitivity of the background *E. coli* strain, and the one including the genomic insert portion of our circuitry to evaluate the baseline tolerance of our constructed strains. We show survival fraction under two gm dosages (Methods; mean and standard deviation, bar, of *N*=24 replicates). **(Bottom)** The cheater/nonproducer strain is also sensitive to gm. Slight differences in survival are expected due to the additional pZS*2R-mCherry plasmid. We assayed its survival to a range of antibiotic concentrations (*N*=12, dots are individual replicates) by following the same protocol as before, but we present here the outcome as surviving colonies per well (with an initial population of ~10^6 cells/well) The rest of the experiments in this paper correspond to a medium (8µg/ml), medium-high (9'5µg/ml) and high (13µg/ml) intensities of antibiotic stress.

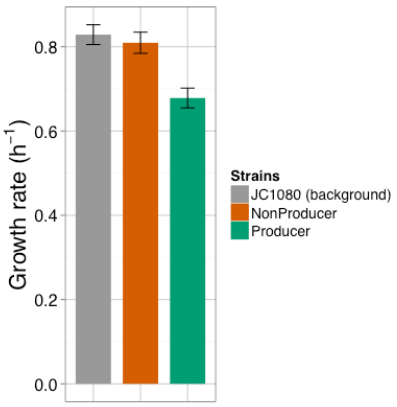

Figure S4. Fitness costs of the genetic circuitry and public good production. The genetic modification of the nonproducer ("cheater") and producer ("cooperator") strains is associated to a slight decrease in growth rate compared to the original strain. The cooperator strain carries an additional cost due to the synthesis of the public good molecule. We calculated these growth rates by obtaining a growth curve (OD_{600} *vs.* time), and extracting the slope from the exponential phase (we plot mean and error of N = 18 replicates). The fitness advantage of cheaters explains their (theoretically expected) invasion of communities of cooperators confirmed in Fig. 2C (main text).

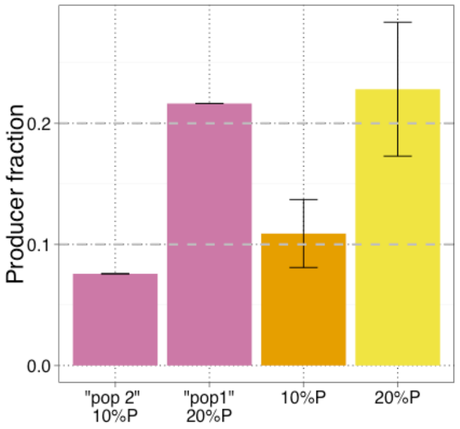

Figure S5. Assembly of bacterial communities with controlled composition. We prepared the initial populations in the different experiments of Fig. 3 by mixing cultures of producers and nonproducers with controlled frequency and concentration as described in the Methods section. We tested the effectiveness of this protocol by directly plating these "designed" populations, and obtaining producer frequency (desired frequency is marked with the grey dashed lines). For experiments associated to "pop 1" and "pop 2" conditions (low population density; pink bars), we calculated producer fraction at the metapopulation level, i.e. combining all the wells in a multiwell plate, while in the initial populations (high density; orange and yellow bars) each well behaves as replica population and the error presented here is associated to individual wells (N=3 wells, each measured 10 times). Overall, we were able to prepare initial populations with sufficient precision. (Note that when assessing the higher initial density conditions additional dilution steps were introduced to allow for CFU counting, so the error provided is an overestimation.)

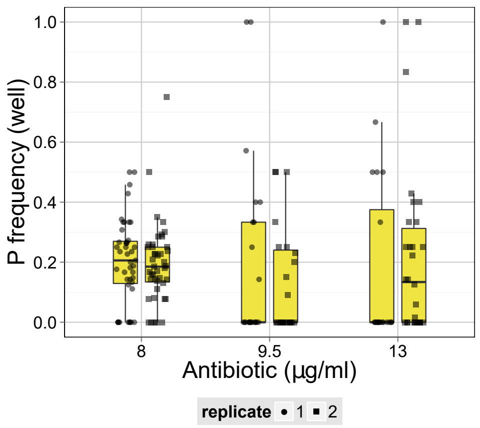

Figure S6. Final cooperator frequency after one round of "accumulation of PG and stress" experiment. In Figs. 3B-C, we present the outcome of one round of accumulation of PG and antibiotic stress on populations with high initial cell density (10^4 cells/well), and varying degrees of invasion by cheaters/nonproducers. The outcome of this first exposure to antibiotic motivates the conditions used in Figs. 3D-E, in terms of both cell density, and producer frequency. We evaluated the change in producer (P) frequency in individual groups after one round of stress for experiments with an initial frequency of 20% producers (Dots and squares indicate the frequency of producers in a single well in the multiwell plate, *N*=45 wells per experimental replicate, 2 experimental replicates. Producer and nonproducer cells were quantified by plating and colony counting and identified by fluorescence color (box plots indicate the median and related statistical parameters). While the outcomes of individual groups are highly variable, the resulting merged metapopulation maintains a producer frequency close to 20% (metapopulation producer frequency = 0.22 for both replicates shown in the figure for gm = 8µg/ml, 0.21 and 0.24 for gm =9.5µg/ml, 0.18 and 0.25 for gm= 13 µg/ml. Strong outliers were excluded from this calculation to reflect general metapopulation behavior. Consequently, we conserved the same P fraction, 20%, for the ensuing experiments.

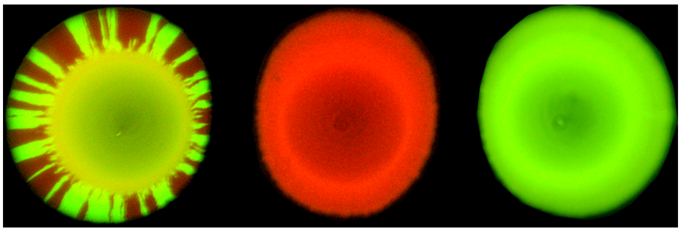

Figure S7. Evaluation of metapopulation composition for experiments with low initial cell density. Plating a 20 µl drop of the culture and allowing it to grow until the outer edges of the colony are visible (~48h) can easily reveal the composition of a population by blue light illumination. When the population is a mix of producers and nonproducers the colony presents a "segmented" pattern with green and red sectors (*left*). When the original culture is composed only of nonproducers or producers the edges of the colony appear as a solid color, respectively, red or green (*right*). A series of founder effects at the edge of the colony give rise to this pattern. Thus, all colonies are organized as a series of clonally expanded sectors, becoming visible only when associated to a distinguishable phenotype; see, for example *[8]* .

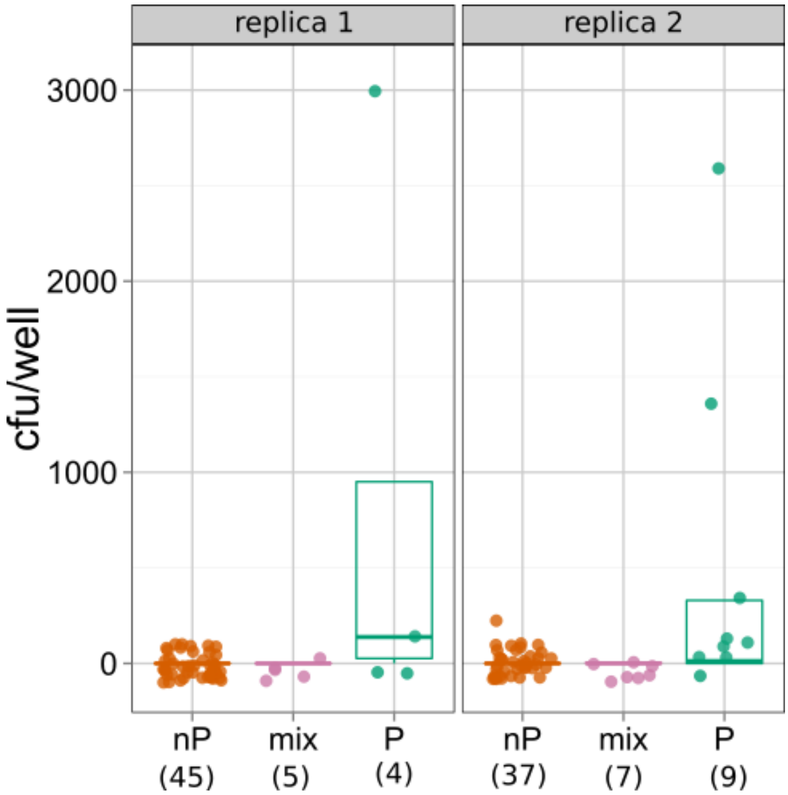

Figure S8. Differential recovery of nP, mixed, and P wells in "pop 2" metapopulations. In the main text, we illustrate experimentally the effect of the ecological Simpson's paradox by characterizing the recovery distribution of a cheater/nonproducer (nP) and a cooperator/producer (P) well under "pop 1" and "pop 2" conditions. Because of the intrinsic variability in the recovery dynamics of any type of well, we should generate many plates with such initial conditions ("pop1" and "pop2"), but this was a very long experimental process to be able to achieve a minimally sound statistical analysis. Thus, the potential fate of metapopulations is best explored by understanding the "typical" behavior of each population composition, instead of the recovery linked to explicit metapopulations. The P and nP distributions then represent extremes of the range of population recovery, with mixed wells expected to present an intermediate behavior (Fig. 3B left panel, main text). We verified this reasoning by evaluating the recovery of 2 replica metapopulations with 20% producers and "pop 2" conditions, and grouping the outcome of individual wells according to composition (the number of wells of each type is indicated below the *x*-axis). As indicated by Fig. 3D (main text), the subpopulations composed of only P recovered disproportionately. Note that mixed wells exhibited recovery rates similar to cheater wells, emphasizing the strength of the ecological Simpson's paradox.

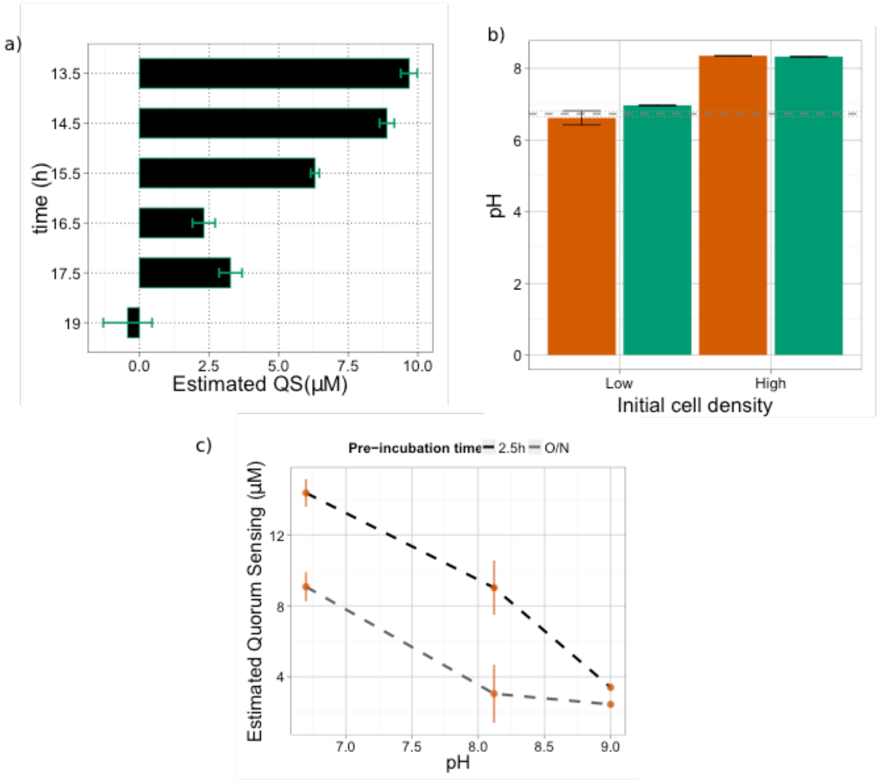

Figure S9. Instability of the public good molecule. (a) As we show in Fig. 4A, accumulation of PG in a culture of producers reaches an upper limit and then starts decaying. Loss of PG associates to the transition into stationary phase. Previous studies (e.g., *[9,10]*) showed that partial degradation of *quorum-sensing* molecules in stationary phase cultures is mediated by an increase in pH (characteristic of bacterial growth in LB due to the consumption of aminoacids). We corroborated this phenomenon in our experimental system: We quantified PG concentration in the medium for cultures of producers in a 96-multiwell plate starting with ~1 cell/well (Fig. 4A-left, main text) or ~10^4 cells/well [panel (a), *N*=~4-5 cells/well]. Accumulation and eventual decay of PG is consistent, starting at earlier times with higher density. **(b)** The pH of the final population is significantly higher, >8 *vs.* ~7, in cultures starting from a comparatively high initial density (of both producers and nonproducers; green and red bars, respectively), while it remains close to the pH of our LB medium (dashed grey line in figure) for cultures that started from a lower density. We measured pH at T_1 (15.5h) by collecting ~35 wells of each type of experiment, removing cells and mineral oil by centrifugation (25min at 4000rpm), and measuring in a pH-meter Basic 20 (Crison, Barcelona, Spain). **(c)** To replicate the effect of pH in a synthetic manner, we added ~10μM of synthetic QS [a concentration that is close to the values estimated in panel (a)] in LB with three different pHs (6.8, 8.1 and 9.1) and incubated for either 2.5h or overnight at 30°C. The pH of the LB medium was adjusted by adding 10N NaOH. Then, we estimated the amount of QS left with the "biosensor" strain following the protocol outlined in the Methods section. We were able to reproduce PG decay.

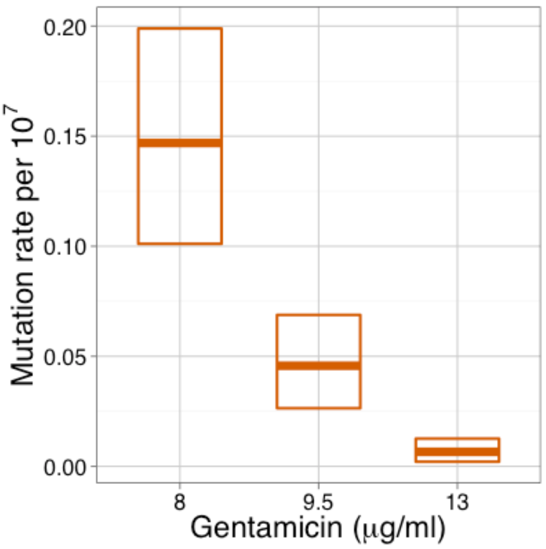

Figure S10. Mutation rates in a range of antibiotic levels. Plating in LB agar plates with the same gm dosage as the one used during the T_2 "stress" period assessed the presence of mutants in the final populations of the "accumulation of PG and stress" experiments. CFUs are then interpreted to be cells with intrinsic resistance to that specific concentration of antibiotic. The results suggest that greater antibiotic stress is associated to a decreased presence of spontaneous mutants, in line with previous work *[11]*. To confirm this observation we computed the mutation rate for the nonproducer strain for the different levels of antibiotic used in the main text: 8μg/ml, 9.5μg/ml, and 13μg/ml (N = 20, the central bar of the crossbars corresponds to the estimated rate, while the edges denote 95% CI; see Methods and *[6]*). Different replica populations were prepared from the same overnight culture, to ensure a shared mutational history. We observed that for a given experiment, the mutation rate decreases with higher antibiotic stress.

Table S1. Oligonucleotides used in this study.

Name	Sequence (5’ → 3’)[a]	Usage / Ref
mCherry-kpnI-F	GG**GGTACC**ATGGTGAGCAAGGGCGAGGAGG	Amplify mCherry adding KpnI-XbaI restriction sites
mCherry-xbaI-R	GC**TCTAGA**TTACTTGTACAGCTCGTCCATG	Amplify mCherry adding KpnI-XbaI restriction sites
Gm-KpnI-F	GG**GGTACC**ATGTTACGCAGCAGCAAC	Amplify Gm^R including the AANDENYALAA peptide tag to the 3´-end and adding KpnI-HindIII restriction sites and diagnose correct integration into *attB*
GmLAA-HindIII-R	CCC**AAGCTT***TTAAGCTGCTAAAGCGTAGTTTTCGTCGTTTGCTGC*GGTGGCGGTACTTGGGTCGATATCAAAGT[b]	Amplify Gm^R including the AANDENYALAA peptide tag to the 3´-end and adding KpnI-HindIII restriction sites
PS1	AGGGCGGCGGATTTGTCC	*[2]*
PS2	GCGGCAACCGAGCGTTC	*[2]*
attB-F	GAGGTACCAGGCGCGGTTTGATC	Diagnose correct integration into *attB*
spec-R	CGAGATCACCAAGGTAGTCGGCA	Diagnose correct integration into *attB*
attB-R	CGATGTTTAGTTAATCACTCTGC	Diagnose correct integration into *attB*
GFP-kpnI-F	GG**GGTACC**ATGAGTAAAGGAGAAGAACTTT	Diagnose correct integration into *attB*

[a] Recognition site for the restriction enzymes specified are indicated in bold in the DNA sequence.
[b] The DNA sequence encoding the peptide AANDENYALAA is shown in italics.

References

1. Chuang JS, *et al.* (2009) Simpson's paradox in a synthetic microbial system. Science 323: 272-275 (2009)

2. Silva-Rocha R, *et al.* (2012) The Standard European Vector Architecture (SEVA): a coherent platform for the analysis and deployment of complex prokaryotic phenotypes. Nucleic Acids Res 41: D666-D675

3. Lutz R, Bujard, H (1997) Independent and tight regulation of transcriptional units in Escherichia coli via the LacR/O, the TetR/O and AraC/I1-I2 regulatory elements Nucleic Acids Res 25: 1203-1210

4. Andersen JB, *et al.* (1998) New unstable variants of green fluorescent protein for studies of transient gene expression in bacteria. Appl Environ Microbiol 64: 2240-2246

5. Keiler KC, Waller PR, Sauer RT (1996) Role of a peptide tagging system in degradation of proteins synthesized from damaged messenger RNA. Science 271: 990-993

6. Foster PL (2006) Methods for determining spontaneous mutation rates. Methods Enzymol, 409: 195-213

7. Cavaliere M, Poyatos JF (2013) Plasticity facilitates sustainable growth in the commons. J R Soc Interface 10: 20121006

8. Van Dyken, JD, Müller MJ, Mack KM, Desai MM. Spatial population expansion promotes the evolution of cooperation in an experimental Prisoner's Dilemma. Curr Biol 23: 919-923

9. Byers JT, Lucas C, Salmond GP, Welch M (2002) Nonenzymatic turnover of an Erwinia carotovora quorum-sensing signaling molecule. J Bacteriol 184: 1163-1171

10. Yates EA, *et al.* (2002) N-acylhomoserine lactones undergo lactonolysis in a pH-, temperature-, and acyl chain length-dependent manner during growth of Yersinia pseudotuberculosis and Pseudomonas aeruginosa. Infect Immun 70: 5635-5646

11. Maclean RC, Buckling, A (2009) The distribution of fitness effects of beneficial mutations in Pseudomonas aeruginosa. PloS Gen 5: e1000406
